# Hexaaqua­magnesium dibromide 5-(pyridinium-3-yl)tetra­zol-1-ide

**DOI:** 10.1107/S1600536810052992

**Published:** 2010-12-24

**Authors:** Jing Dai, Xin-Yuan Chen

**Affiliations:** a Ordered Matter Science Research Center, College of Chemistry and Chemical Engineering, Southeast University, Nanjing 210096, People’s Republic of China

## Abstract

In the title compound, [Mg(H_2_O)_6_]Br_2_·2C_6_H_5_N_5_, the Mg^II^ atom, lying on an inversion center, is coordinated by six water mol­ecules in a distorted octa­hedral geometry. The pyridine and tetra­zole rings in the 5-(pyridinium-3-yl)tetra­zol-1-ide zwitterion are nearly coplanar, twisted from each other by a dihedral angle of 5.70 (1)°. The zwitterions, Br anions and complex cations are connected by O—H⋯Br, O—H⋯N and N—H⋯Br hydrogen bonds, leading to the formation of a three-dimensional network.

## Related literature

For tetra­zole derivatives, see: Fu *et al.* (2008 ▶); Zhao *et al.* (2008 ▶). For the crystal structures and properties of related compounds, see: Fu *et al.* (2007 ▶, 2009 ▶); Fu & Xiong (2008 ▶).
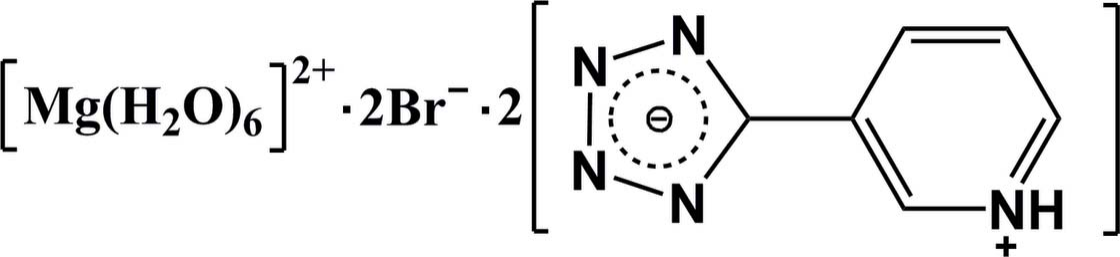


## Experimental

### 

#### Crystal data

[Mg(H_2_O)_6_]Br_2_·2C_6_H_5_N_5_
*M* *_r_* = 586.53
Triclinic, 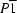

*a* = 7.3439 (15) Å
*b* = 8.7786 (18) Å
*c* = 9.5863 (19) Å
α = 94.04 (3)°
β = 90.94 (3)°
γ = 111.75 (3)°
*V* = 572.0 (2) Å^3^
*Z* = 1
Mo *K*α radiation
μ = 3.62 mm^−1^
*T* = 298 K
0.40 × 0.05 × 0.05 mm

#### Data collection

Rigaku SCXmini CCD diffractometer
Absorption correction: multi-scan (*CrystalClear*; Rigaku, 2005 ▶) *T*_min_ = 0.89, *T*_max_ = 0.95
5933 measured reflections
2627 independent reflections
2172 reflections with *I* > 2σ(*I*)
*R*_int_ = 0.040

#### Refinement

*R*[*F* ^2^ > 2σ(*F* ^2^)] = 0.040
*wR*(*F* ^2^) = 0.097
*S* = 1.09
2627 reflections
142 parameters
H-atom parameters constrained
Δρ_max_ = 0.32 e Å^−3^
Δρ_min_ = −0.52 e Å^−3^

### 

Data collection: *CrystalClear* (Rigaku, 2005 ▶); cell refinement: *CrystalClear*; data reduction: *CrystalClear*; program(s) used to solve structure: *SHELXTL* (Sheldrick, 2008 ▶); program(s) used to refine structure: *SHELXTL*; molecular graphics: *SHELXTL* and *DIAMOND* (Brandenburg, 1999 ▶); software used to prepare material for publication: *SHELXTL*.

## Supplementary Material

Crystal structure: contains datablocks I, global. DOI: 10.1107/S1600536810052992/hy2389sup1.cif

Structure factors: contains datablocks I. DOI: 10.1107/S1600536810052992/hy2389Isup2.hkl

Additional supplementary materials:  crystallographic information; 3D view; checkCIF report

## Figures and Tables

**Table 1:** Hydrogen-bond geometry (Å, °)

*D*—H⋯*A*	*D*—H	H⋯*A*	*D*⋯*A*	*D*—H⋯*A*
N1—H1*A*⋯Br1^i^	0.86	2.41	3.240 (3)	161
O1*W*—H1*WA*⋯N5	0.81	1.98	2.780 (3)	171
O1*W*—H1*WB*⋯Br1^ii^	0.89	2.66	3.382 (3)	138
O2*W*—H2*WA*⋯N4	0.86	1.89	2.738 (3)	167
O2*W*—H2*WB*⋯Br1^iii^	0.76	2.53	3.296 (2)	178
O3*W*—H3*WA*⋯Br1^iv^	0.91	2.48	3.328 (2)	156
O3*W*—H3*WB*⋯N2^v^	0.96	1.78	2.730 (3)	174

Symmetry codes: (i) 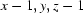; (ii) 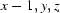; (iii) 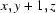; (iv) 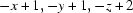; (v) 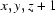.

**Fig. 1.**
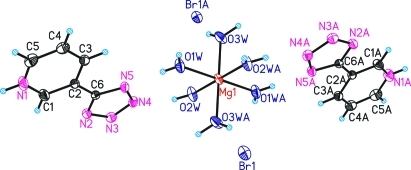
Molecular structure of the title compound. Displacement ellipsoids are drawn at the 30% probability level. [Symmetry code: (A) 1 - x, 1 - y, 2 - z.]

**Fig. 2.**
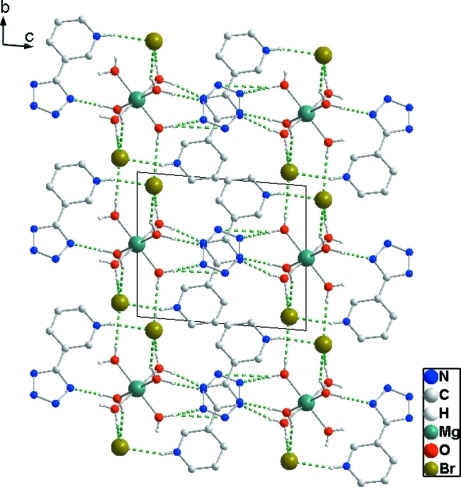
The crystal packing of the title compound. H atoms not involved in hydrogen bonds (dashed lines) have been omitted for clarity.

## supplementary crystallographic information

### Comment

Tetrazole compounds have attracted more attention as phase transition dielectric materials for its applications in micro-electronics, memory storage. With the purpose of obtaining phase transition crystals of 3-(1*H*-tetrazol-5-yl)pyridine compounds, its interaction with various metal ions has been studied and a series of new materials have been elaborated with this organic molecule (Fu *et al.*, 2007, 2008; Fu & Xiong 2008; Zhao *et al.*, 2008). In this paper, we describe the crystal structure of the title compound.

The asymmetric unit of the title compound (Fig. 1) is composed of one zwitterionic organic molecule, half [Mg(H_2_O)_6_]^2+^ cation and one Br anion. In the zwitterionic organic molecule, the pyridine N atom is protonated. The pyridine and tetrazole rings are nearly coplanar and only twisted from each other by a dihedral angle of 5.70 (1)°. The geometric parameters of the tetrazole ring are comparable to those in related molecules (Fu *et al.*, 2009; Zhao *et al.*, 2008).

In the crystal structure, the intermolecular hydrogen bonds are formed by all H atoms of the water molecules and pyridine N atoms with the tetrazole N atoms or Br anions. The complex cations [Mg(H_2_O)_6_]^2+^ and Br anions are linked in the crystal through O—H···Br hydrogen bonds into an infinite cation–anion sheet parallel to (0 0 1). The two-dimensional sheets are linked by organic molecules through O—H···N and N—H···Br hydrogen bonds into a three-dimensional network (Table 1 and Fig. 2).

### Experimental

MgBr_2_.6H_2_O (2 mmol) and 3-(1*H*-tetrazol-5-yl)pyridine (0.528 g, 2 mmol) were dissolved in 70% methanol aqueous solution, and then 2 ml HBr was added. Single crystals suitable for X-ray diffraction analysis were obtained by slow evaporation of the solution at room temperature after two weeks. The crystals were colourless, block, and of average size 0.2×0.3×0.4 mm.

The permittivity measurement shows that there is no phase transition within temperature range from 100 to 400 K, and the permittivity is 9.1 at 1 MHz at room temperature.

### Refinement

H atoms attached to C and N atoms were positioned geometrically and treated as riding, with C—H = 0.93 and N—H = 0.86 Å and with *U*_iso_(H) = 1.2*U*_eq_(C, N). H atoms of water molecules were located in a difference Fourier map and refined using a riding model, with *U*_iso_(H) = 1.5*U*_eq_(O).

### Crystal data

**Table:**

[Mg(H_2_O)_6_]Br_2_·2C_6_H_5_N_5_	*Z* = 1
*M**_r_* = 586.53	*F*(000) = 294
Triclinic, *P*1	*D*_x_ = 1.703 Mg m^−^^3^
Hall symbol: -P 1	Mo *K*α radiation, λ = 0.71073 Å
*a* = 7.3439 (15) Å	Cell parameters from 2627 reflections
*b* = 8.7786 (18) Å	θ = 3.1–24.5°
*c* = 9.5863 (19) Å	µ = 3.62 mm^−^^1^
α = 94.04 (3)°	*T* = 298 K
β = 90.94 (3)°	Block, colourless
γ = 111.75 (3)°	0.40 × 0.05 × 0.05 mm
*V* = 572.0 (2) Å^3^	

### Data collection

**Table:**

Rigaku SCXmini CCD diffractometer	2627 independent reflections
Radiation source: fine-focus sealed tube	2172 reflections with *I* > 2σ(*I*)
graphite	*R*_int_ = 0.040
Detector resolution: 13.6612 pixels mm^-1^	θ_max_ = 27.5°, θ_min_ = 3.1°
ω scans	*h* = −9→9
Absorption correction: multi-scan (*CrystalClear*; Rigaku, 2005)	*k* = −11→11
*T*_min_ = 0.89, *T*_max_ = 0.95	*l* = −12→12
5933 measured reflections	

### Refinement

**Table:**

Refinement on *F*^2^	Primary atom site location: structure-invariant direct methods
Least-squares matrix: full	Secondary atom site location: difference Fourier map
*R*[*F*^2^ > 2σ(*F*^2^)] = 0.040	Hydrogen site location: inferred from neighbouring sites
*wR*(*F*^2^) = 0.097	H-atom parameters constrained
*S* = 1.09	*w* = 1/[σ^2^(*F*_o_^2^) + (0.0426*P*)^2^ + 0.1011*P*] where *P* = (*F*_o_^2^ + 2*F*_c_^2^)/3
2627 reflections	(Δ/σ)_max_ = 0.001
142 parameters	Δρ_max_ = 0.32 e Å^−^^3^
0 restraints	Δρ_min_ = −0.52 e Å^−^^3^

### Fractional atomic coordinates and isotropic or equivalent isotropic displacement parameters (Å^2^)

**Table:**

	*x*	*y*	*z*	*U*_iso_*/*U*_eq_	
N4	0.3716 (3)	0.6009 (3)	0.6076 (3)	0.0347 (6)	
N2	0.2637 (3)	0.5318 (3)	0.3932 (2)	0.0315 (5)	
C6	0.1639 (4)	0.4135 (3)	0.4740 (3)	0.0263 (6)	
N5	0.2266 (3)	0.4528 (3)	0.6073 (2)	0.0328 (5)	
C2	0.0038 (4)	0.2612 (3)	0.4229 (3)	0.0272 (6)	
C3	−0.1073 (4)	0.1506 (4)	0.5142 (3)	0.0319 (6)	
H3	−0.0792	0.1730	0.6101	0.038*	
N1	−0.1923 (4)	0.0847 (4)	0.2374 (3)	0.0481 (7)	
H1A	−0.2202	0.0642	0.1489	0.058*	
N3	0.3947 (3)	0.6479 (3)	0.4800 (3)	0.0360 (6)	
C1	−0.0427 (5)	0.2234 (4)	0.2822 (3)	0.0398 (7)	
H1	0.0296	0.2941	0.2180	0.048*	
C5	−0.3000 (5)	−0.0234 (4)	0.3232 (4)	0.0464 (8)	
H5	−0.4017	−0.1188	0.2873	0.056*	
C4	−0.2598 (4)	0.0073 (4)	0.4639 (4)	0.0408 (7)	
H4	−0.3335	−0.0668	0.5253	0.049*	
Mg1	0.5000	0.5000	1.0000	0.0303 (3)	
O1W	0.2634 (3)	0.3471 (3)	0.8692 (2)	0.0497 (6)	
H1WA	0.2453	0.3674	0.7906	0.075*	
H1WB	0.1806	0.2467	0.8849	0.075*	
O2W	0.5507 (3)	0.6851 (2)	0.8697 (2)	0.0465 (6)	
H2WA	0.5117	0.6602	0.7829	0.070*	
H2WB	0.6098	0.7777	0.8780	0.070*	
O3W	0.3123 (4)	0.5786 (3)	1.1155 (2)	0.0532 (6)	
H3WA	0.2760	0.6585	1.0834	0.080*	
H3WB	0.3030	0.5606	1.2130	0.080*	
Br1	0.79518 (5)	0.08632 (4)	0.89951 (3)	0.04481 (14)	

### Atomic displacement parameters (Å^2^)

**Table:**

	*U*^11^	*U*^22^	*U*^33^	*U*^12^	*U*^13^	*U*^23^
N4	0.0315 (12)	0.0309 (13)	0.0353 (14)	0.0055 (11)	−0.0054 (10)	−0.0025 (11)
N2	0.0307 (12)	0.0285 (12)	0.0293 (13)	0.0041 (10)	0.0010 (10)	0.0027 (10)
C6	0.0242 (13)	0.0277 (14)	0.0257 (14)	0.0080 (11)	0.0012 (10)	0.0028 (11)
N5	0.0325 (12)	0.0336 (13)	0.0262 (13)	0.0053 (11)	−0.0038 (10)	0.0027 (10)
C2	0.0270 (13)	0.0270 (14)	0.0269 (14)	0.0097 (11)	0.0010 (11)	0.0009 (11)
C3	0.0309 (14)	0.0311 (15)	0.0313 (15)	0.0083 (12)	0.0010 (11)	0.0051 (12)
N1	0.0513 (16)	0.0464 (17)	0.0333 (15)	0.0059 (14)	−0.0096 (12)	−0.0124 (13)
N3	0.0332 (13)	0.0292 (13)	0.0411 (15)	0.0063 (11)	0.0026 (11)	0.0039 (11)
C1	0.0410 (16)	0.0376 (17)	0.0297 (16)	0.0025 (14)	0.0027 (13)	−0.0010 (13)
C5	0.0369 (17)	0.0301 (17)	0.061 (2)	0.0016 (14)	−0.0041 (16)	−0.0084 (16)
C4	0.0344 (16)	0.0307 (16)	0.052 (2)	0.0049 (13)	0.0049 (14)	0.0061 (15)
Mg1	0.0387 (7)	0.0245 (7)	0.0216 (7)	0.0047 (6)	0.0004 (5)	0.0022 (5)
O1W	0.0520 (13)	0.0454 (14)	0.0302 (12)	−0.0069 (11)	−0.0088 (10)	0.0073 (10)
O2W	0.0710 (15)	0.0213 (10)	0.0310 (12)	−0.0015 (10)	−0.0103 (10)	0.0041 (9)
O3W	0.0792 (17)	0.0629 (17)	0.0342 (13)	0.0432 (15)	0.0173 (12)	0.0161 (12)
Br1	0.0546 (2)	0.03047 (19)	0.0402 (2)	0.00473 (15)	0.00206 (14)	0.00592 (14)

### Geometric parameters (Å, °)

**Table:**

N4—N3	1.312 (4)	C5—H5	0.9300
N4—N5	1.341 (3)	C4—H4	0.9300
N2—N3	1.336 (3)	Mg1—O2W^i^	2.048 (2)
N2—C6	1.336 (3)	Mg1—O2W	2.048 (2)
C6—N5	1.329 (3)	Mg1—O3W^i^	2.061 (2)
C6—C2	1.460 (4)	Mg1—O3W	2.061 (2)
C2—C1	1.373 (4)	Mg1—O1W	2.087 (2)
C2—C3	1.390 (4)	Mg1—O1W^i^	2.087 (2)
C3—C4	1.386 (4)	O1W—H1WA	0.8068
C3—H3	0.9300	O1W—H1WB	0.8907
N1—C5	1.333 (4)	O2W—H2WA	0.8615
N1—C1	1.339 (4)	O2W—H2WB	0.7636
N1—H1A	0.8600	O3W—H3WA	0.9085
C1—H1	0.9300	O3W—H3WB	0.9576
C5—C4	1.363 (5)		
			
N3—N4—N5	110.0 (2)	O2W^i^—Mg1—O2W	180.000 (1)
N3—N2—C6	105.2 (2)	O2W^i^—Mg1—O3W^i^	91.93 (10)
N5—C6—N2	111.4 (2)	O2W—Mg1—O3W^i^	88.07 (10)
N5—C6—C2	124.2 (2)	O2W^i^—Mg1—O3W	88.07 (10)
N2—C6—C2	124.4 (2)	O2W—Mg1—O3W	91.93 (10)
C6—N5—N4	104.5 (2)	O3W^i^—Mg1—O3W	180.000 (1)
C1—C2—C3	117.8 (3)	O2W^i^—Mg1—O1W	89.55 (9)
C1—C2—C6	120.7 (3)	O2W—Mg1—O1W	90.45 (9)
C3—C2—C6	121.5 (3)	O3W^i^—Mg1—O1W	90.10 (11)
C4—C3—C2	120.7 (3)	O3W—Mg1—O1W	89.90 (11)
C4—C3—H3	119.6	O2W^i^—Mg1—O1W^i^	90.45 (9)
C2—C3—H3	119.6	O2W—Mg1—O1W^i^	89.55 (9)
C5—N1—C1	123.3 (3)	O3W^i^—Mg1—O1W^i^	89.90 (11)
C5—N1—H1A	118.3	O3W—Mg1—O1W^i^	90.10 (11)
C1—N1—H1A	118.3	O1W—Mg1—O1W^i^	180.000 (1)
N4—N3—N2	108.8 (2)	Mg1—O1W—H1WA	122.5
N1—C1—C2	119.9 (3)	Mg1—O1W—H1WB	126.6
N1—C1—H1	120.1	H1WA—O1W—H1WB	110.2
C2—C1—H1	120.1	Mg1—O2W—H2WA	119.0
N1—C5—C4	119.4 (3)	Mg1—O2W—H2WB	133.7
N1—C5—H5	120.3	H2WA—O2W—H2WB	107.0
C4—C5—H5	120.3	Mg1—O3W—H3WA	118.6
C5—C4—C3	119.0 (3)	Mg1—O3W—H3WB	118.7
C5—C4—H4	120.5	H3WA—O3W—H3WB	119.2
C3—C4—H4	120.5		

Symmetry codes: (i) −*x*+1, −*y*+1, −*z*+2.

### Hydrogen-bond geometry (Å, °)

**Table:**

*D*—H···*A*	*D*—H	H···*A*	*D*···*A*	*D*—H···*A*
N1—H1A···Br1^ii^	0.86	2.41	3.240 (3)	161
O1W—H1WA···N5	0.81	1.98	2.780 (3)	171
O1W—H1WB···Br1^iii^	0.89	2.66	3.382 (3)	138
O2W—H2WA···N4	0.86	1.89	2.738 (3)	167
O2W—H2WB···Br1^iv^	0.76	2.53	3.296 (2)	178
O3W—H3WA···Br1^i^	0.91	2.48	3.328 (2)	156
O3W—H3WB···N2^v^	0.96	1.78	2.730 (3)	174

Symmetry codes: (ii) *x*−1, *y*, *z*−1; (iii) *x*−1, *y*, *z*; (iv) *x*, *y*+1, *z*; (i) −*x*+1, −*y*+1, −*z*+2; (v) *x*, *y*, *z*+1.
